# Validated LC-MS/MS Assays for Novel Triazolyl-Oxazolidinones with Anticonvulsant Activities and Their Potential Applications in Stability and Pharmacokinetic Studies

**DOI:** 10.3390/ph19071005

**Published:** 2026-06-28

**Authors:** Mohsen A. Hedaya, Oludotun A. Phillips, Vidhya Thomas, Mohammad G. Qaddoumi, Samuel B. Kombian, Naser F. Al-Tannak

**Affiliations:** 1 Department of Pharmaceutics, College of Pharmacy, Kuwait University, Sabah Al Salem University City, Safat, 13060 Shadadiya, Kuwait; vidhyaelizabeth@gmail.com; 2 Department of Pharmaceutical Chemistry, College of Pharmacy, Kuwait University, Sabah Al Salem University City, Safat, 13060 Shadadiya, Kuwait; oludotun.phillips@ku.edu.kw (O.A.P.); dr.altannak@ku.edu.kw (N.F.A.-T.); 3 Department of Pharmacology and Therapeutics, College of Pharmacy, Kuwait University, Sabah Al Salem University City, Safat, 13060 Shadadiya, Kuwait; mohammad.qaddoumi@ku.edu.kw; 4 Department of Pharmacology & Toxicology, School of Pharmacy and Pharmaceutical Sciences, University for Development Studies, P.O. Box TL 1350 Tamale, Ghana; skombian@uds.edu.gh

**Keywords:** oxazolidinones, LC-MS/MS, anticonvulsants, method validation, stability, pharmacokinetics, tissue distribution

## Abstract

**Background**: We recently synthesized a novel series of triazolyl-oxazolidinones (PH066, PH139, PH162, and PH166) that exhibited significant in vivo anticonvulsant activity in rat models of electrically- and chemically-induced seizures. **Objectives**: The objectives of the current study were to develop and validate analytical UPLC-MS/MS assays for quantitative determination of these novel compounds in plasma and tissue samples obtained from pharmacokinetic studies to support and/or explain the reported in vivo effects in rats, as well as to study the stability of these compounds in the solid state. **Methods**: Sensitive, selective, precise, and accurate UPLC-MS/MS methods were developed using a UPLC BEH C_18_ column for analyte separation. The mobile phase consisted of water with 0.1% formic acid and acetonitrile with 0.1% formic acid in different ratios depending on the analyte of interest. Quantitative determination was performed using the multiple-reaction monitoring scanning mode. The methods were utilized to determine the concentration of the four compounds in plasma and tissue samples obtained at different time points after intraperitoneal (IP) injection of 100 mg/kg of each compound to rats. The stability of the compounds was investigated under forced degradation conditions. **Results**: The developed methods were specific for each of the analytes and were found to be linear in the concentration range of 5–30 μg/mL in plasma and 0.5–25 μg/g in tissue samples. The intra-day and inter-day precision and accuracies were within the acceptable range for the four compounds. The methods were applied to quantify these compounds in the plasma and various tissue samples obtained from rats after IP administration of 100 mg/kg of each compound. The maximum plasma concentrations were 14.6, 9.20, 14.5, and 18.1 μg/mL, and those of brain were 2.6, 0.22, 1.9, and 2.8 μg/g for PH066, PH139, PH162, and PH166, respectively. The compounds degraded under forced degradation conditions to various degradation products. **Conclusions**: The developed methods were selective, linear, accurate, and precise and are suitable for quantitation of the four compounds in plasma and tissue samples obtained during pharmacokinetic investigations, as well as for detecting and quantifying their degradation products.

## 1. Introduction

Oxazolidinone is a class of five-membered heterocyclic compounds that have been shown to have several pharmacological activities. Linezolid is the first compound in this class that was approved by the U.S. Food and Drug Administration (FDA) in 2000 for treating Gram-positive-resistant bacterial infections [1,2,3]. In addition to their antibacterial effects [4,5,6], over the past 20 years, oxazolidinones have demonstrated other potential pharmacological effects, such as antitubercular [7,8,9], anticancer [10,11], anti-inflammatory [12,13], antiviral [14], and anticoagulant [15], as well as having effects in neurological [16,17] and metabolic disorders [18,19,20]. Efforts to discover potential antiseizure agents with activity covering all seizure types [21,22] have led to the identification of compounds with pharmacophoric structures that are different from current antiseizure drugs [23]. These include compounds that belong to the oxazolidinone class [24,25,26,27].

Investigation of PH192 (Table 1), a triazolyl-oxazolidinone derivative with good safety and efficacy profiles, demonstrated dose-dependent protection of mice and rats from electrically- and chemically-induced seizures. Intraperitoneal administration of 100 mg/kg PH192 protected 75% of mice and 66–83% of rats from electrically-induced seizures and 80% of the rats from pentylenetetrazol-induced seizures for only 30 min [28]. The observed anticonvulsant effect of PH192 in both seizure models in rats encouraged us to investigate the anticonvulsant activity of other triazolyl-oxazolidinone derivatives to identify useful lead compounds for further development as drug candidates. More recently, we investigated the anticonvulsant activity of four triazolyl-oxazolidinone derivatives—PH066, PH139, PH162, and PH166 (Table 1)—in comparison to the previously studied PH192 against electrically- and chemically-induced seizures in rats. The results showed that the selected triazolyl-oxazolidinone derivatives protected at least 20–100% of rats from electrically- and chemically-induced seizures tested at 120 min after IP administration. The order of the anticonvulsant efficacy and duration for all of the investigated compounds was as follows: PH162 > PH139 > PH166 ≈ PH066 > PH192 [29]. The differences in the activities and duration of action can, plausibly, be attributed to differences in the pharmacokinetic properties of these compounds. This may include differences in their systemic bioavailability after IP administration, differences in the extent of brain distribution, the possibility of the formation of pharmacologically active metabolites, and differences in the elimination rate of the compounds. It is, therefore, important to consider the pharmacokinetic characteristics of these compounds when investigating their anticonvulsant activity.

Performing quantitative pharmacokinetic studies requires the development of accurate, precise, fast, and sensitive analytical assays for each compound. The scientific literature is replete with various analytical techniques useful for assessing the bioavailability and stability of drugs and investigational compounds containing oxazolidinone heterocycle [30,31,32,33,34,35,36,37,38,39,40,41]. Most of these reported techniques utilized different instrumental high-performance liquid chromatography (HPLC) coupled with ultraviolet-visible detectors (UV-Vis) or mass spectrometer detectors, using different mobile and stationary phases. Only a few of these methods were developed for the analysis of oxazolidinone derivatives in plasma, and only one of these methods was applied to tissue distribution and pharmacokinetic studies [41]. Our objective here was to develop analytical procedures to be applied in stability and pharmacokinetic studies of the four novel compounds.

We hereby report the development and validation of UPLC-MS/MS assays for four novel triazolyl-oxazolidinone derivatives (PH066, PH139, PH162, and PH166, shown in Table 1), which have previously demonstrated potent and efficacious anticonvulsant activity. Forced degradation studies of the compounds under acidic, basic, and oxidative environments are also presented. The developed analytical assays were applied to study the stability of the compounds and to determine the concentrations of each compound in plasma and tissue samples obtained from pilot pharmacokinetic studies in rats. These assays will be useful in quantifying the concentrations of the investigated compounds in rat plasma and brain tissues and correlating these with their observed in vivo anticonvulsant effects.

## 2. Results

### 2.1. Assay Validation

#### 2.1.1. Selectivity

**Figure 1:**
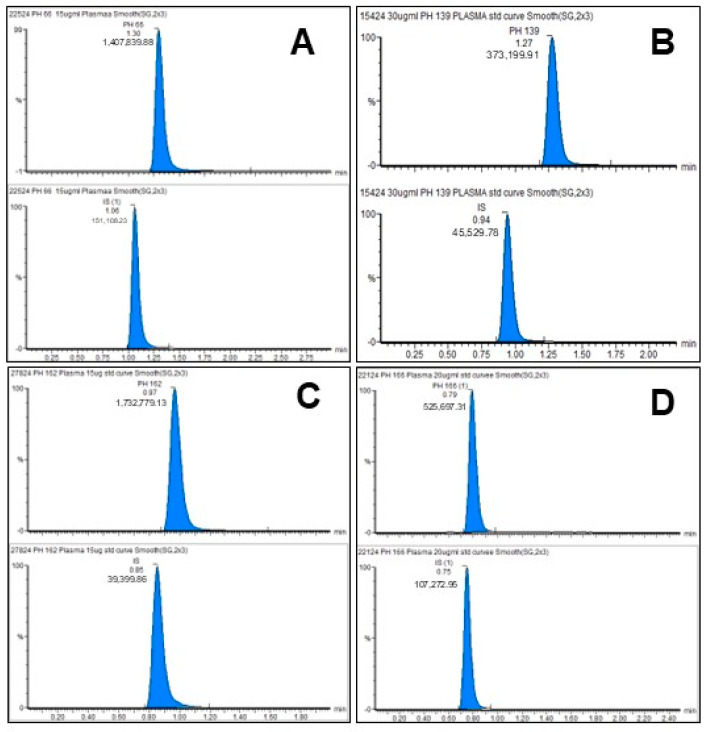
Representative chromatograms for PH066 and IS at 1.30 and 1.06 m (**A**), PH139 and IS at 1.27 and 0.94 m (**B**), PH162 and IS at 0.97 and 0.85 m (**C**), and PH166 and IS at 0.79 and 0.75 m (**D**), respectively.

None of the developed methods showed interference from any of the endogenous compounds present in the plasma or brain-tissue homogenates. Figure 1 shows representative examples of the chromatograms of the compounds under investigation and the IS in the plasma samples.

#### 2.1.2. Linearity and Sensitivity

The assays were linear in the range of 5–30 µg/mL in rat plasma and 0.1–5 μg/mL in tissue homogenate, which is equivalent to 0.5–25 µg/g in tissues. The average coefficients of determination for the obtained linear regression equations calculated using the data analysis program were 0.993, 0.990, 0.992, and 0.992 for the plasma standard curves and were 0.998, 0.996, 0.994, and 0.998 for the brain tissue standard curves for PH066, PH139, PH162, and PH166, respectively. Also, the calculated residuals were randomly distributed around the straight line. The estimated LOQs in plasma were 7.0, 15, 11, and 14 ng/mL, while the estimated LODs were 2, 5, 3, and 4 ng/mL, for PH066, PH139, PH162, and PH166, respectively. There were no carryovers from one sample to the other, as demonstrated by the absence of any compounds detected during the analysis of blank plasma- and blank brain-tissue homogenates after the analysis of the calibration standards.

#### 2.1.3. The Matrix Effect

There were no significant difference between the predicted concentrations of the quality control samples prepared in the plasma- and in brain-tissue homogenates using the plasma and the tissue homogenate standard curves with CVs of 3.4, 7.1, 4.4, and 3.8% for PH066, PH139, PH162, and PH166, respectively.

#### 2.1.4. Precision and Accuracy

The ranges of the assay precisions determined from CV% values of the assay responses for the three sets of the calibration standards at three different concentrations were 0.97–10.2%, 0.66–3.31%, 0.38–7.73%, and 6.13–7.81% for the intra-day precision and 2.33–5.96%, 0.85–8.45%, 2.29–8.60% and 1.09–9.27% for the inter-day precision for PH066, PH139, PH162, and PH166, respectively. The ranges of accuracy determined from the percent deviation from the nominal concentrations of the three sets of standards at the three different concentrations were 96.3–108%, 94.7–104%, 95.3–109%, and 98.0–105%, while those for the inter-day accuracies were 97.3–107%, 98.7–105%, 95.7–109%, and 98.0–109%, for PH066, PH139, PH162, and PH166, respectively. The detailed precision and accuracy data are presented in Table 2. The variations in the slopes of the linear regression equations for the six different standard curves obtained over a period of 1 month for each compound were all below 10% of the mean value, indicating the methods’ reproducibility.

#### 2.1.5. Stability During Analysis

The concentrations of PH066, PH139, PH162, and PH166 determined at time 0 and at 24 h, when kept at room temperature, did not change by more than 4.0% for all of the compounds, indicating their stability at room temperature over 24 h. Also, the concentrations predicted during the analysis of the frozen quality-control samples (5, 10, and 30 μg/mL), prepared in rat plasma over a period of 3 weeks, were in the range of 4.1–8.0%, 3.2–6.1%, 2.4–5.1%, and 3.5–7.3% for PH066, PH139, PH162, and PH166, respectively, indicating good stability when frozen at −80 °C.

#### 2.1.6. Extraction Efficiency

The average extraction efficiencies for PH066, PH139, PH162, PH166, and the internal standard from rat plasma determined at concentrations of 10 µg/mL and 30 µg/mL were 90.7, 70.1, 75.9, 40.9, and 50.3, respectively. Table 3 presents detailed results of the determination of the extraction efficiencies of the compounds under investigation.

### 2.2. Stability Studies

Table 4, Table 5 and Table 6 provide summaries of the degradation products of PH066, PH139, PH162, and PH166 after exposure to forced acidic, basic, and oxidative degradation at 90 °C for 90 min.

### 2.3. Preliminary Pharmacokinetics of the Novel Triazolyl-Oxazolidinone Derivatives in Rats

**Figure 2:**
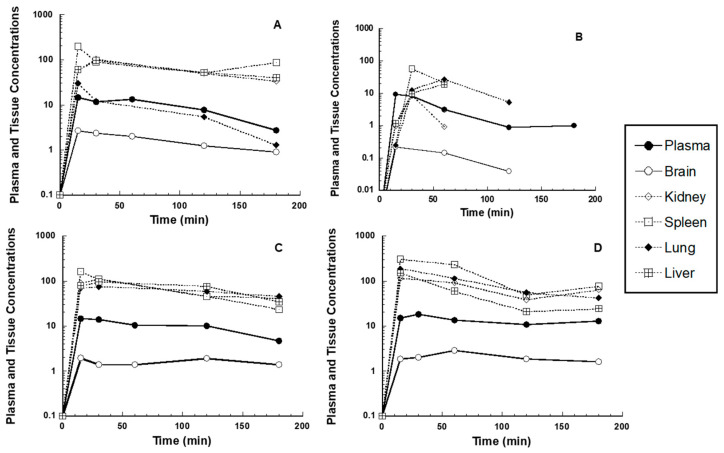
Graphs showing the concentrations in plasma (μg/mL) and different tissues (μg/g) of PH066 (**A**), PH139 (**B**), PH162 (**C**), and PH166 (**D**) after IP administration of 100 mg/kg of each compounds to rats.

The maximum plasma and tissue concentrations of the four compounds were achieved in most tissues during the first 30 min after IP administration. The maximum plasma concentrations were 14.6, 9.2, 14.5, and 18.1 μg/mL, and the maximum brain tissue concentrations were 2.6, 0.22, 1.9, and 2.8 μg/g for PH066, PH139, PH162, and PH166, respectively. The brain tissue concentrations of PH066, PH162, and PH166 were about 15–20% of their concentrations in plasma, while their concentrations in the kidney, spleen, lung, and liver tissues were several folds higher than their plasma concentrations. Furthermore, the concentrations in plasma and tissues declined over the three-hour experiment period, with the fastest rate of decline being observed in the following order: PH139 > PH066 > PH162 > PH166. The brain and other tissue concentrations of PH139 were much lower than the tissue concentrations of the other compounds, which may have resulted from the rapid decline in plasma concentrations of PH139. Figure 2 represents the measured plasma and tissue concentrations at different time points after IP administration of the four compounds in rats using the above analytical assays.

## 3. Discussion

The main objectives of the current investigations were to develop validated analytical methods for quantitative determination of four novel triazolyl-oxazolidinone derivatives in biological fluids/tissues and to investigate the stability of these compounds under forced degradation conditions. These compounds have previously demonstrated anticonvulsant activity against electrically- and chemically-induced seizure models in rats. Our future plan is to utilize these methods in the analysis of plasma- and brain-tissue samples obtained at different time points after administration of these novel compounds in rats for concurrent evaluation and correlation of their pharmacokinetic behavior and pharmacodynamic (anticonvulsant) behavior.

These analytical methods are crucial in experiments that aim to characterize drug dose–concentration–effect relationships. Determination of the concentration of a drug or an investigational compound at the target end organ (site of action) can differentiate between the absence of a pharmacodynamic effect due to ineffectiveness (e.g., due to the lack of an intrinsic activity or inappropriate target interactions) and limited bioavailability of the pharmacological agent at the site of action. Similarly, determination of the concentration of the pharmacological agent at the site of action when pharmacological activity is observed is important in the characterization of the pharmacodynamic model that can best describe the drug concentration–effect relationship [42].

Analytical methods have been developed for other triazolyl-oxazolidinone derivatives. However, these methods were not used for the determination of the compounds’ concentrations in biological samples after their administration [30,31,32,33,34,35,36,37,38,39,40]. Only one method was developed and applied for the determination of linezolid and two novel oxazolidinone derivatives with potential antibacterial activity to study their pharmacokinetics in rabbits to explain the discrepancies in their in vitro and in vivo antibacterial activities [41,43]. The methods developed in the current study were applied to determine the concentrations of the compounds under investigation in plasma and tissue samples obtained at different time points after their IP administration in rats. Although the compounds are related in their chemical structure, they have different molecular and physicochemical characteristics, which necessitate modification of the chromatographic and detection parameters for optimization of the analytical methods for each individual compound.

Drug regulatory authorities have developed guidelines for the validation of analytical methods that should be followed in the drug development processes [44,45]. When we applied these guidelines to the developed methods, it was clear that these methods were specific for each analyte, with no interference from any endogenous compounds in plasma or tissue samples, as demonstrated in Figure 1. The methods were linear based on the linear regression analysis of the relationship between the analyte/IS peak area ratio and the compound’s concentration in the range of concentrations 5.0–30.0 μg/mL in plasma and 0.5–25.0 μg/g in tissue samples. This range of concentrations in plasma and tissues covers the range of the compounds’ concentrations in plasma and tissues observed after administration to rats in doses that produced anticonvulsant activities, as indicated in the results (Figure 2). Variation in the intra-day and inter-day precisions of the analytical methods for the four compounds at three different concentrations for each compound were below 10%, indicating that the developed methods were quite precise. The same was true for the determined intra-day and inter-day accuracy, where the differences between the measured concentrations and the nominal concentrations were all in the acceptable range (Table 2). The extraction efficiencies for the four compounds and the IS were different due to the differences in their physicochemical properties. However, the extraction efficiencies were not different for the different concentrations within each compound (Table 3). The validation results indicate that the developed methods were in concert with the guidelines of the international regulatory authorities [44,45].

The developed methods were applied in the analysis of plasma and tissue samples obtained from a preliminary pharmacokinetic study after IP administration of each compound in rats. The range of the observed plasma and tissue sample concentrations of the investigated compounds were, in most cases, within the range of the developed methods, indicating that the developed methods can be used for the analysis of samples obtained in future extensive pharmacokinetic studies of these novel compounds as anticonvulsants in rats. The quantification of each compound in the brain will be used to correlate with their anticonvulsant activities.

Forced degradation studies were performed to investigate the stability of PH066, PH139, PH162, and PH166 under acid (1 N HCl), basic (1 N NaOH), and oxidative (1 N H_2_O_2_) conditions at 90 °C for 90 min. Stability studies conducted under these forced degradation conditions are essential for the development of formulations and the determination of the shelf-life of any potential drug candidate. Different compounds gave different degradation products under different forced conditions. For example, under 1 N hydrochloric acid conditions, PH066, PH166, and PH162 underwent amide bond hydrolysis to produce the deacylated piperazine degradant M + 1 = 347 for PH066 and PH166 and M + 1 = 361 for PH162. Similar amide bond hydrolysis under acidic conditions was reported for some oxazolidinone derivatives containing the amide functionality including linezolid [37,38,39]. In this study, some PH162 (M + 1 = 510) remained undegraded, while PH139 was found to be relatively stable with no breakdown product observed under acidic conditions. The underlying reason for this remains unclear; however, both PH139 and PH162 possess nitro substituents on the phenyl and the furyl moieties, respectively (Table 4), which may have contributed to their observed stability. Amides generally undergo hydrolysis (breakage of the C-N bond by water) under acidic or basic conditions, which leads to the formation of carboxylic acids and amines. Under basic experimental conditions, the hydroxide ion (OH^−^) acts as a nucleophile, which attacks the carbonyl carbon of the amide, forming an intermediate anion. This is followed by an elimination step, where the amine is displaced, followed by deprotonation of the carboxylic acid intermediate by the amine. Therefore, strong electron-withdrawing groups, if rightly placed, are expected to make the carbonyl carbon more electropositive, which will facilitate the amide hydrolysis reaction under basic conditions. Moreover, under acidic experimental conditions, hydrolysis of amide proceeds firstly by protonation of the oxygen of the carbonyl group, which will increase the electrophilicity of carbonyl carbon. This is followed by attack of a water molecule to form the tetrahedral intermediate, which eventually collapses, via breakage of the carbon–nitrogen bond. This results in the formation of carboxylic acid and the amine [46].

However, under acidic experimental conditions electron-withdrawing groups, if in direct interaction with the carbonyl carbon, can make it harder for protonation of the carbonyl oxygen. This can indirectly hinder the progress of the amide bond hydrolysis. This is probably why compounds PH139 and PH162, containing the 5-nitrofuryl and 3-nitrophenyl moieties, respectively, were found to be relatively more stable under acidic hydrolysis conditions than the other derivatives.

By contrast, under basic (1 N sodium hydroxide) environment, all the four compounds PH066, PH162, PH166 and PH139 had degradation products. Three of them—PH066, PH162 and PH139—underwent base-promoted amide bond hydrolysis coupled with the hydrolysis of the oxazolidinone ring to produce the deacylated piperazine-ring-opened degradant M + 1 = 321 for PH066 and M + 1 = 335 for both PH162 and PH139. In contrast, PH166 had two degradation products, which resulted from the base-promoted amide bond hydrolysis to produce the deacylated piperazine-ring-opened degradant M + 1 = 347 and from the adduct of the sodium addition to PH166, M + Na + 1 = 530, to yield PH166-Na. Such ring-opened degradants have been reported to be responsible for the instability of linezolid [37,39], sutezolid [40], and tedizolid [46]. Similar to PH166, a sodium-adduct degradation product, M + Na = 532, was also observed, yielding PH162-Na (Table 5).

Finally, under oxidative-stress conditions, after adding 1 mL of hydrogen peroxide (1 N H_2_O_2_) and heating at 90 °C for 90 min, PH066, PH162, and PH166 had one degradation product each, while PH139 was relatively stable with no degradation product detected. The products of oxidation may have resulted from the N-oxidation of the piperazine nitrogen in the structure (Table 6). Similar oxidation of nitrogen-containing heterocycles has been reported for linezolid in H_2_O_2_ [39,47]. These results suggest that other than PH139, the other three compounds may be relatively more unstable when formulated in acid or basic formulations and/or when exposed to air. These findings may be important to consider in the preparation and storage of these compounds. Additional thorough standard stability studies of potential lead compound(s) will be necessary to completely characterize the stability of these compounds during research or future dosage formulations processes.

## 4. Materials and Methods

### 4.1. Chemical Synthesis

The triazolyl-oxazolidinone derivatives under investigation are novel compounds that are not available commercially. PH066, PH139, PH162, PH166, and the internal standard (IS), shown in Table 1, were synthesized, purified, and characterized using a previously published methodology [48,49,50]. The morpholine-containing 5-hydroxymethyl-oxazolidinone compound was used as the IS in this analysis. All of the derivatives were purified and fully characterized by analytical methods, including 1H NMR, 13C NMR (Bruker Avance II 600 NMR spectrometer, Bruker Corporation, Billerica, MA, USA), mass spectrometry (Finnigan MAT INCOS XL mass spectrometer, Thermo Fisher Scientific, Waltham, MA, USA), infrared (IR, Perkin Elmer System 2000 FT-IR spectrometer, PerkinElmer, Shelton, CT, USA), and elemental analyses (LECO elemental analyzer CHNS 932 apparatus, LECO Corporation, St Joseph, MI, USA). All of the equipment was located at the Research Sector Projects Unit (RSPU) at the College of Science, Kuwait University.

### 4.2. Instrumentation

Quantitative determination of the concentrations of the triazolyl-oxazolidinone derivatives in the plasma and tissue samples was accomplished using LC-MS/MS methods. Although the investigated oxazolidinone derivatives are related in their chemical structures, each derivative required different analytical procedures and instrumental conditions because of the differences in the chemical structures and physicochemical properties. Sample analysis was performed using Waters Acquity UPLC H-Class Xevo TQD system (Waters Corporation, Milford, MA, USA) consisting of a quaternary pump, an auto sampler, a column oven, and a TQ mass spectrometer (MS/MS) equipped with an electrospray ionization probe operated in the positive ionization mode (+ESI). MassLynx software version 4.2 (Waters Corporation, MA, USA)was used for data processing and reporting. Chromatographic separation of analytes was carried out on an Acquity UPLC BEH C_18_ (50 mm × 2.1 mm, 1.7 um) column connected to a Van Guard Pre-column.

#### 4.2.1. Ultra-Pressure Liquid Chromatography

Isocratic elution was carried out using a Waters Acquity UPLC system with quaternary solvent manager. The mobile phase consisted of filtered and degassed 0.1% formic acid in water and 0.1% formic acid in acetonitrile in various ratios for the different compounds, as indicated in Table 7.

#### 4.2.2. Mass Spectroscopy

The mass spectroscopy (MS) parameters were determined manually by injecting 500 ppb of a sample directly into the mass spectrometer. Then optimization of the various parameters was achieved by an automatic instrument tuning method (Intelistart) to obtain the multiple-reaction monitoring (MRM) conditions with the highest sensitivity for detecting parent and daughter ion masses for all of the compounds and the IS. The optimized compound-dependent parameters and the source-dependent parameters are listed in Table 8. Quantitative determination was performed using the MRM scanning mode of the parent to daughter mass ions of *m*/*z*, 441.2700 → 95.0300 for PH066, 500.2800 → 209.0500 for PH139, 510.2027 → 150.0203 for PH162, 508.2000 → 347.1300 for PH166, and 297.2200 → 163.1700 for the IS, respectively.

### 4.3. Pharmacokinetic Study of the Novel Triazolyl-Oxazolidinone Derivatives in Rats

A pilot pharmacokinetic study, including absorption and tissue distribution of the compounds under investigation was performed in male Sprague–Dawley (SD) rats obtained from and housed in the HSC Animal Resource Center, Kuwait University. All animal experiments were approved by Kuwait University Health Sciences Center Animal Research Ethics committee. The experiments were performed in accordance with the guidelines for the care and use of experimental animals established by the Canadian Council on Animal Care. For each of the four compounds under investigations, 5 different male SD rats (100–150 g) were used. Rats in each group received 100 mg/kg IP of each compound dissolved in DMSO. Rats were euthanized with an overdose of halothane at 15, 30, 60, 120, and 180 min after administration of each compound. Blood, brain, liver, lung, spleen, and kidneys were harvested immediately. Plasma was obtained by centrifugation (1500× *g* for 10 min) of harvested blood, while tissue samples were harvested and weighed. All samples were kept at −80 °C until analysis.

#### Preparation of Calibration Standards and Sample in Plasma and Tissues

The calibration standards were prepared using blank rat plasma or blank tissue homogenates. The tissue homogenates were prepared by homogenization of tissues in a volume 5 times their weight of phosphate buffer, pH 7.0. An aliquot of 200 µL blank rat plasma or blank tissue homogenate was spiked with 100 µL of the working solutions of compounds to produce the calibration standards in the concentration range 5–30 µg/mL in plasma and 0.1–5 μg/mL in tissue homogenate (equivalent to 0.5–25 µg/g tissue). The standard samples were mixed with 30 µL of 10 ng/mL methanolic solution of the IS. The mixture was vortexed for 1 min and then extracted with 600 µL of diethyl ether and centrifuged at 13,200 rpm for 10 min. The ether layer was transferred into another tube and evaporated to dryness under the flow of nitrogen gas. The residue was reconstituted with 300 µL of mobile phase, filtered through a 0.22 μm syringe filter, and then 10 µL of the filtrate was injected onto the UPLC-MS/MS. When plasma/tissue samples with unknown concentrations were analyzed, 200 µL of sample was mixed with 100 µL of the blank solvent and 30 µL of IS solution and the resulting mixture was extracted and processed the same way as that of the calibration standards.

### 4.4. Analytical Method Validation

Interpretation of the data obtained from pharmacokinetic experiments is dependent on the validity of the measured concentrations of the compounds in plasma and tissue samples obtained during the experiments. Therefore, validation of the developed analytical method is important and should involve assessment of selectivity, linearity, accuracy, precision, and extraction efficiency, all required by regulatory authorities [44,45]. Assay validation was performed for the analysis of the rat plasma samples containing the compounds under investigation. For the analysis of tissue samples obtained from the pilot pharmacokinetics experiments, brain-tissue homogenates were used to prepare the calibration standards used to determine concentrations in all tissue samples.

#### 4.4.1. Selectivity

The selectivity of a method is the ability of the method to accurately measure a specific analyte within a complex mixture or matrix without interference from other components. The selectivity was determined by analyzing blank rat plasma- and brain-tissue homogenates obtained from the rats used in the pharmacokinetics experiments.

#### 4.4.2. Sensitivity

The sensitivity of the assay was determined from the lower limit of quantitation (LOQ), which is the lowest concentration that can be quantified with acceptable coefficient of variation (CV), usually ≤15%. While the lower limit of determination (LOD) is the lowest concentration that can be detected in the chromatograms (usually taken as 3 times the baseline noise).

#### 4.4.3. Linearity

Calibration curves were constructed from the relationship of the analyte/IS peak area ratio (PAR) and the analyte concentration in the calibration standards using the least-square linear regression analysis. Linearity was evaluated by examining the coefficient of determination (R^2^) of the straight-line equations representing the calibration curves and by examining the residuals, which is the difference between the nominal and the predicted analyte concentrations.

#### 4.4.4. The Matrix Effect

For each compound, two sets of quality control samples (*n* = 5, each) containing 5 μg/mL in plasma and in tissue homogenate. The matrix effect was evaluated by comparing the predicted concentrations in the two sets of quality control samples, using the plasma and the tissue homogenate calibration curves.

#### 4.4.5. Precision and Accuracy

Intra-day precision and accuracy were determined by analyzing 3 sets of calibration standards; each set included 3 different concentrations, for each compound on the same day. The CV in the assay response (PAR) for each concentration was used as a measure of precision at different concentrations. While the difference between the nominal concentration and the back-calculated concentrations for each calibration standard was used as a measure of assay accuracy. Inter-day accuracy and precision were determined in the same way, but the 3 sets of calibration standards at three different concentrations were analyzed in three different days. The assay reproducibility was assessed by examining the slopes of 6 different curves analyzed on 6 different days over a period of one month.

#### 4.4.6. Stability During Analysis

The stability of PH066, PH139, PH162, and PH166 in the mobile phase at room temperature for 24 h was determined by spiking 300 mL of the mobile phase with the compounds’ working solutions to produce concentrations of 5, 10, and 30 μg/mL of each compound in triplicate and then adding 30 μL of internal standard solution. For each compound, the concentrations measured at time 0.00 and after 24 h were compared to determine the 24 h stability at room temperature. To study the stability during the freeze–thaw cycle, blank rat plasma was used to prepare quality control samples containing 5, 10, and 30 μg/mL of each compound. These samples were frozen and then analyzed as unknown samples in duplicates during three different runs over a period of 3 weeks. The predicted concentrations during the different runs were then compared.

#### 4.4.7. Extraction Efficiency

The absolute extraction efficiencies of each of the four compounds and IS from rat plasma were determined at concentrations of 10 and 30 μg/mL. Two sets of standards were prepared, one in the mobile phase and the other in rat plasma. The standard solutions in the mobile phase, which represent 100% recovery, were injected directly into the UPLC-MS/MS, while the plasma standards underwent all the extraction procedures before injection. The absolute peak heights for each compound in the un-extracted and extracted samples were compared to determine the absolute extraction efficiency for each compound.

### 4.5. Forced Degradation Studies

All stability tests below were conducted on the compounds under investigation in the solid state, which may reflect the conditions under which any future dosage form(s) may be exposed.

#### 4.5.1. Acidic Degradation

Two milligrams (2 mg) of each of the compounds (PH066, PH139, PH166, and PH162) were weighed and placed in a 4 mL vial, separately. Two milliliters of 1 N HCl were added to each compound and heated at 90 °C for 90 min, allowed to cool down for 15 min, and then an aliquot of 100 µL of each mixture was mixed with 900 µL acetonitrile. The degradation product(s) were detected using LC-MS/MS (Xevo G2-S QToF, Waters Corporation, MA, USA).

#### 4.5.2. Basic Degradation

Similar to the acid degradation experiments above, two milligrams of each compound was weighed and placed in a 4 mL vial, separately. The compounds were subjected to basic stress conditions by adding 2 mL of 1 N NaOH and heated at 90 °C for 90 min, allowed to cool down for 15 min, and then an aliquot of 100 µL of each mixture was mixed with 900 µL acetonitrile and the degradation product(s) were detected using LC- MS/MS (Xevo G2-S QToF).

#### 4.5.3. Oxidation Degradation

Finally, for the oxidative stress test, 2 mg of each compound was weighed and placed in a 4 mL vial. The compounds were subjected to oxidative conditions by adding 2 mL of 1 N H_2_O_2_. The samples were then heated at 90 °C for 90 min, allowed to cool down for 15 min, and an aliquot of 100 µL of each mixture was mixed with 900 µL acetonitrile and the degradation product(s) were detected using LC- MS/MS (Xevo G2-S QToF).

## 5. Conclusions

In conclusion, the developed UPLC-MS/MS methods for the quantitative determination of PH066, PH139, PH162, and PH166 in plasma and tissues were sensitive, specific, accurate, and reproducible within instrumental and regulatory guideline limits. The methods are, therefore, suitable for measuring the concentrations of the compounds in plasma and tissue samples obtained after administration of these compounds to animals (rats).

## Figures and Tables

**Table 1:** Triazolyl-oxazolidinone derivatives investigated in this study.

Code	Structures	Mol Form.	Mol wt.	CLogP ^a^
**PH066**	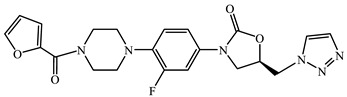	C_21_H_21_FN_6_O_4_	440.4354	0.6088
**PH139**	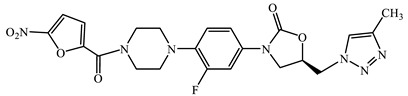	C_22_H_22_FN_7_O_6_	499.4594	0.7828
**PH162**	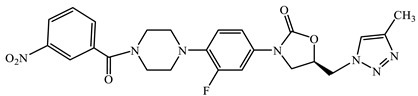	C_24_H_24_FN_7_O_5_	509.4984	1.607
**PH166**	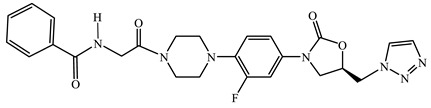	C_25_H_2_6FN_7_O_4_	507.5264	1.217
**PH192**	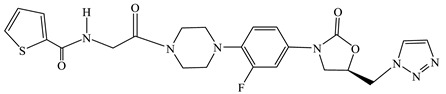	C_23_H_24_FN_7_O_4_S	513.5484	1.055
**Internal Standard (IS)**	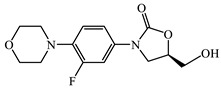	C_14_H_17_FN_2_O_4_	296.2984	0.2360

^a^ Calculated Log of partition coefficient (cLogP) values were computed using PerkinElmer ChemDraw Professional Version 12.1.21, PerkinElmer Informatics, Inc., Shelton, Connecticut (CT), USA, 1998–2020.

**Table 2:** Inter-day and intra-day accuracy and precision.

Compound	Nominal Concentrations (μg/mL)	Measured Concentrations (μg/mL) (Mean + S.D.)	Precision CV (%)	Accuracy (%)
A- Intra-Day				
PH066	10	10.8 ± 1.10	10.2	108
	15	15.2 ± 1.12	7.37	101
	30	28.9 ± 0.28	0.97	96.3
PH139	10	10.4 ± 0.20	1.92	104
	15	14.2 ± 0.47	3.31	94.7
	30	30.4 ± 0.20	0.66	101
PH162	10	9.53 ± 0.61	6.40	95.3
	15	16.3 ± 1.26	7.73	109
	30	28.9 ± 0.11	0.38	96.3
PH166	10	10.5 ± 0.82	7.81	105
	15	14.7 ± 0.91	6.19	98.0
	30	31.0 ± 1.90	6.13	103
B- Inter-Day				
PH066	10	10.7 ± 0.30	2.80	107
	15	14.6 ± 0.87	5.96	97.3
	30	29.6 ± 0.69	2.33	98.7
PH139	10	10.5 ± 0.28	2.67	105
	15	14.8 ± 1.25	8.45	98.7
	30	30.5 ± 0.26	0.85	102
PH162	10	10.9 ± 0.25	2.29	109
	15	16.4 ± 1.41	8.60	109
	30	28.7 ± 0.90	3.14	95.7
PH166	10	10.9 ± 1.01	9.27	109
	15	14.7 ± 0.16	1.09	98.0
	30	30.5 ± 1.50	4.92	102

**Table 3:** The extraction efficiencies for the four compounds and IS.

Compound	Concentration μg/mL	Extraction Efficiency (%)	Average Extraction Efficiency (%)
PH066	10	91.9	90.7
PH066	30	89.8	
PH139	10	72.7	70.1
PH139	30	67.5	
PH162	10	79.5	75.9
PH162	30	72.4	
PH166	10	49.1	40.9
PH166	30	32.7	
Internal Standard	0.3	50.2	50.3

**Table 4:** Forced degradation studies’ results for compounds PH066, PH139, PH162, and PH166 under acidic (1N HCl) conditions at 90 °C for 90 min.

Compound	Degradant Chemical Structure	Mass Spectrum
PH066 (molecular weight: 440.4354 g/mol)	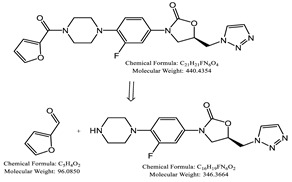	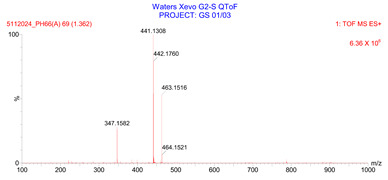
PH166 (molecular weight: 507.5264 g/mol)	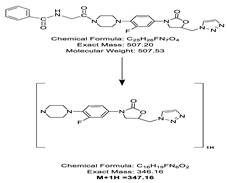	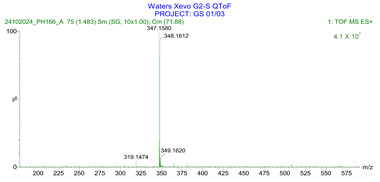
PH162 (molecular weight: 509.4984 g/mol)	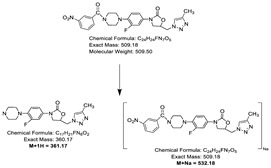	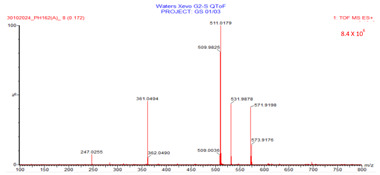
PH139 (molecular weight: 499.4594 g/mol)	No degradation observed Only the starting PH139 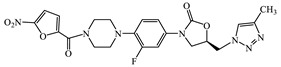	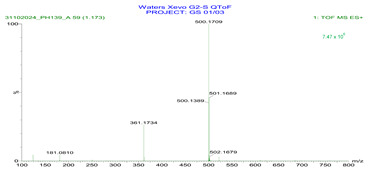

**Table 5:** Forced degradation studies’ results for compounds PH066, PH139, PH162, and PH166 under basic (1N NaOH) conditions at 90 °C for 90 min.

Compound	Degradant Chemical Structure	Mass Spectrum
PH066 (molecular weight: 440.4354 g/mol)	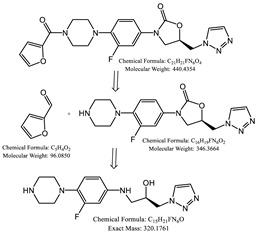	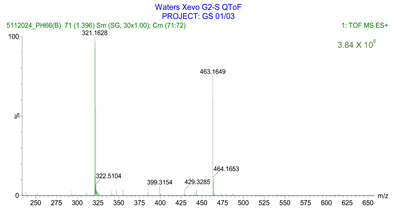
PH166 (molecular weight: 507.5264 g/mol)	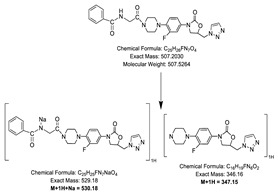	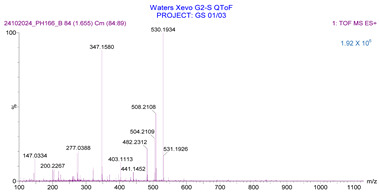
PH162 (molecular weight: 509.4984 g/mol)	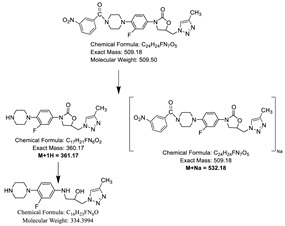	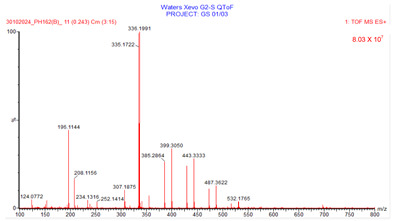
PH139 (molecular weight: 499.4594 g/mol)	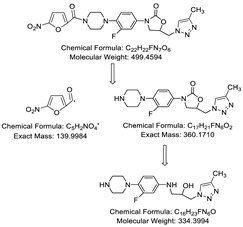	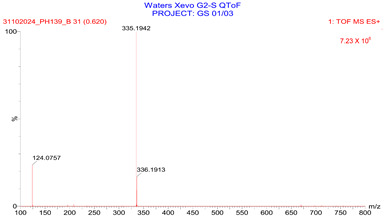

**Table 6:** Forced degradation studies’ results for compounds PH066, PH139, PH162, and PH166 under oxidation (1 N H_2_O_2_) conditions at 90 °C for 90 min.

Compound	Degradant Chemical Structure	Mass Spectrum
PH066 (molecular weight: 440.4354 g/mol)	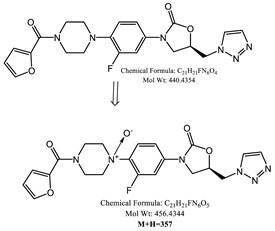	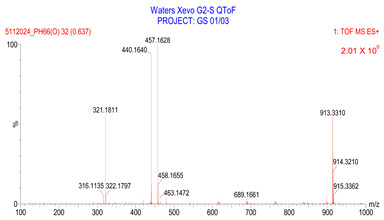
PH166 (molecular weight: 507.5264 g/mol)	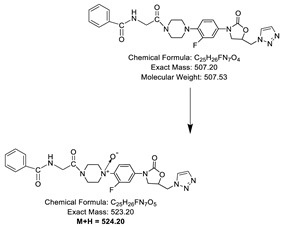	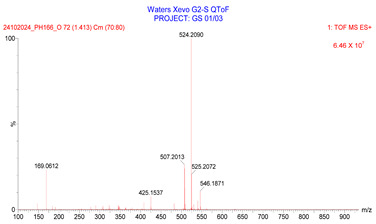
PH162 (molecular weight: 509.4984 g/mol)	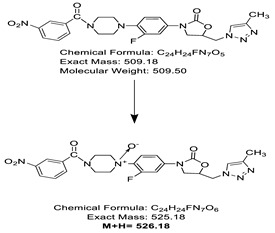	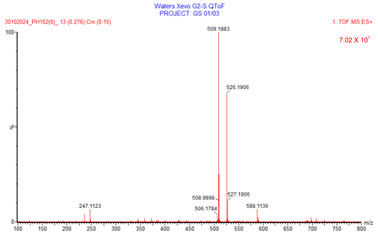
PH139 (molecular weight: 499.4594 g/mol)	No degradation was observed	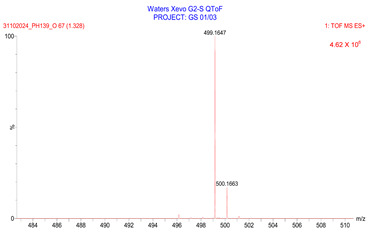

**Table 7:** Chromatographic conditions for the four investigated compounds.

Compound Name	Mobile Phase A (%)	Mobile Phase B (%)	Flow Rate (ml/min)	Column Temp (°C)	Sample Temp. (°C)
PH066	60	40	0.200	35	off
PH139	50	50	0.200	30	off
PH162	40	60	0.200	off	5
PH166	50	50	0.250	26	off

**Table 8:** The LC-MS/MS parameters for the tested compounds.

Compound	Compound-Dependent Parameters				Source-Dependent Parameters	
	Source Desolvation Temp (°C)	Capillary Source Voltage (kV)	Cone Voltage (V)	Collision Energy (V)	Desolvation Gas Flow (l/h)	Cone Gas Flow (l/h)
PH066	500	0.50	46	80	1000	0
PH139	200	3.60	65	30	650	0
PH162	200	3.40	64	42	650	0
PH166	450	3.50	50	30	650	0
IS	300	3.50	42	25	800	50

## Data Availability

The data presented in this study are openly available in [KU Research Sector] at [https://www.ku.edu.kw/research], accessed on 24 June 2026.
